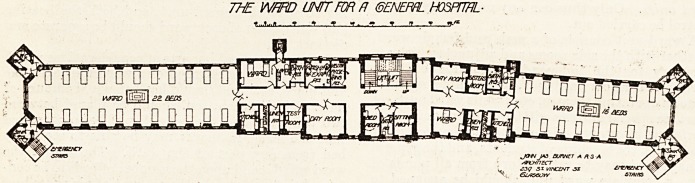# The Units of General Hospital Construction

**Published:** 1907-05-04

**Authors:** 


### HOSPITAL ADMINISTRATION.

### CONSTRUCTION AND ECONOMICS.

### THE UNITS OF GENERAL HOSPITAL CONSTRUCTION.

#### I. THE WARD UNIT.

Having dealt in detail with the admission department and the various conditions governing its efficient management, the hospital proper next claims attention. By this term is understood that part of the hospital buildings in which the patients are accommodated. The provision therein made should be based upon a definite scheme or plan of *ward units*. Only thus can any uniform system of control be carried out.

A ward unit consists of a male and female ward, together with the side rooms and adjuncts which are necessary for the independent working of these wards. A surgical ward unit, for example, must include an operating theatre, etc., while ward units set apart for special diseases will be modified to meet any special requirements. In order that the treatment of patients, the teaching of students, and the general management may be satisfactory from all points of view, a complete ward unit should be assigned to each member of the visiting staff—medical, surgical, or special, as the case may be. Each of these units, so far as the structure of the buildings is concerned, should be complete in itself, and its constituent parts should all be on the same floor of the building. The ward units in a general hospital may be multiplied according to the available area of site, the requirements of the district which is to be served, and the funds available for upkeep. They should, however, be limited to what can be efficiently supervised by one executive officer, and conveniently managed from a central administrative department.

Medical and surgical ward units, while they have many points in common, differ essentially in others. We shall deal first with the medical unit.

Each visiting physician should have an average of 40 beds, and not more than 44, under his charge, 20 to 26 for males and 16 to 18 for females. This accommodation should be divided as follows:—

A male ward containing 18 to 22 beds.

A small side ward with two beds for males.

A female ward containing 16 to 18 beds.

A small side ward with one or two beds for females.

There are certain adjuncts which must be in close proximity to every ward; these include a ward-kitchen and small scullery, a linen room, a day room for convalescents, a bathroom and lavatory, a sanitary tower for w.c.'s, and a sanitary tower for bed-pan washer and urine rack. Each of these apartments will be described in detail. In addition to these, the ward unit should include (*a*) a private room for the visiting physician, where he may keep the records of his cases and interview his assistants; (*b*) a well-lighted room for research work, such as the testing of urine, microscopical examinations, etc., fitted with a sink, hot and cold water, and a centrifuge; (*c*) a duty room for the sister in charge of the ward unit; (*d*) lavatory accommodation for the nursing staff apart from that provided for patients; (*e*) house physician's quarters. This latter should include sitting-room, bedroom, and bathroom, and should be as near his wards as possible, but completely cut off from them.

A cross-ventilating passage should separate the wards from each other and from the staircase leading to any other ward unit, and both the heating and ventilation of each ward should be separate and distinct.

There are several important considerations which must govern the designing of a ward unit. In the first place, each bed should have an equal amount of light and air; each, too, should be placed between two windows, and the beds should be so arranged that the nurse in charge may be able to see every bed from any part of the ward. The size of the ward will depend upon the number of beds to be contained therein, and on the amount of air-space and floor-space allowed for each patient. In a hospital with a medical school attached the amount of floor-space per bed, and cubic space per patient, must of necessity be greater than are considered sufficient for a hospital without such a school; the fact that the major part of the clinical instruction is given in the wards renders such increased provision highly desirable. In a teaching school a minimum of 2,000 cubic feet of air-space should be allowed per patient, and a minimum of 125 square feet of floor-space per bed.

The windows of the ward should be 4 feet wide, should be placed 3 feet from the floor, and should extend to the ceiling, so that there is no unventilated area where foul air may remain above the windows. Probably the best form of ward window yet devised is the double-sash window with hopper top, and guarded on both sides so that no direct draught strikes down on the patients. The sash frames should open inwards to facilitate cleaning and should be double glazed, with a space of half-an-inch between the inner and outer panes to prevent radiation and loss of heat. The window-sills should be plate-glass slabs, 14 inches wide, resting on the window-ledge behind and on an angle iron bar in front.

The walls should be tiled to a height of 6 feet 6 inches, and above this treated with cement or adamant plaster. They should be rounded on to the roof so that there may be no angles, the walls and ceiling forming one continuous surface. The plastered walls and ceiling, after being thoroughly dry, should be treated with three coats of oil paint and two of enamel.

Floors should be fire-proof, and covered with a hard wood such as teak, maple, or pitch-pine. Three-inch boards of red pine tongued and grooved, laid on concrete and covered with tongued and grooved teak parquetry, make an excellent ward floor. The wood must be well stoved before being laid, and afterwards polished with a mixture of beeswax and turpentine. The junction of floor and wall should be a curve and be formed in terrazzo. This terrazzo should extend 1 foot up the walls to meet the tiles, and 18 inches into the floor-area. It should also be carried round all stoves and radiators 2 feet at the sides, and 4 feet 6 inches in front, thus preventing the possibility of sparks falling on the wood floor, a point frequently overlooked in hospital construction.

All doors should be of teak and should present an absolutely smooth surface without panels or mouldings. Those at the entrance to the ward should be capable of being opened both outwards and inwards, and should be of plain teak, the upper section being fitted with plate glass.

The accompanying plan of the new pavilion of the Glasgow Western Infirmary affords a comprehensive idea of a complete ward unit. This ward is all comprised on one flat and runs north and south. It consists of a male ward with 22 beds and a female ward with 16 beds; a side ward for males with two beds and a side ward for females with one bed. A central corridor joins the two large wards, and this corridor is reached by a central staircase or by two electric automatic lifts, which are placed in the well of the stair. The female ward is on the north side, and at its northern end are two sanitary towers completely separated from the ward by means of cut-off ventilating passages. These passages are protected by louvred doors on either side, which may be opened or closed at will. In the west tower are two w.c.'s, and these are separated from each other by a brick partition which extends to the roof. This complete partition is much more satisfactory than the method of division by a screen 6 or 8 feet high, which is so often adopted. Each w.c. has two windows, so that quite independently of the cut-off passage which separates them from the ward, either can be ventilated as desired. In the east tower is an apartment for the exclusive use of the nurse, and here provision is made for cleansing bed-pans, storing urines, etc.

Adjoining the ward, and on the west side of the main corridor, is a lavatory for patients, and abutting on this is a private lavatory for nurses. Leading from the patients' lavatory is the ward bathroom, but we leave their appurtenances with others to be subsequently described in detail and illustrated. The next apartment is the sister's duty room, and beyond it the day-room for the use of patients. Adjoining the ward on the east side of the main corridor is the ward kitchen; the cleaners' room, linen room, and side ward are placed in the order named.

A through ventilating corridor running east and west cuts off this ward and its adjuncts from the remainder of the ward unit, and a similar short corridor occupies a corresponding position at the end of the male ward and its adjuncts. Between those two cross corridors is the central staircase before mentioned, and opposite to it the private quarters of the resident physician. These comprise sitting-room, bedroom, and bathroom.

THE WARD UNIT FOR A GENERAL HOSPITAL.

The male ward, like that for women, has at its southern end two towers. The apartments adjoining it are also similar to those of the female ward, with the addition of a urine-test room, and a room for washing and examining patients. There is also a private room for the visiting physician, which is entered from the short cut-off corridor.

(*To be continued.*)

## Figures and Tables

**Figure f1:**